# USP5 knockdown alleviates lung cancer progression via activating PARP1-mediated mTOR signaling pathway

**DOI:** 10.1186/s13062-023-00371-z

**Published:** 2023-04-14

**Authors:** Lei Cao, Hongsheng Liu, Cheng Huang, Chao Guo, Luo Zhao, Chao Gao, Yuan Xu, Guige Wang, Naixin Liang, Shanqing Li

**Affiliations:** Department of Thoracic Surgery, Peking Union Medical College, Peking Union Medical College Hospital, Chinese Academy of Medical Sciences, No. 1, Shuai Fu Yuan, Dong Cheng District, Beijing, 100730 China

**Keywords:** USP5, mTOR, PARP1, Lung cancer

## Abstract

**Background:**

With the rapidly increasing morbidity and mortality, lung cancer has been considered one of the serious malignant tumors, affecting millions of patients globally. Currently, the pathogenesis of lung cancer remains unclear, hindering the development of effective treatment. This study aims to investigate the mechanisms of lung cancer and develop an effective therapeutic approach for intervention in preventing lung cancer progress.

**Methods:**

The USP5 levels are detected in lung cancerous and paracancerous tissue by quantitative real-time polymerase chain reaction (qRT-PCR) and Western blotting methods to explore their roles in lung cancer progression. MTT, colony assay, and transwell chamber approaches are employed to measure cell viability, proliferation, and migration, respectively. Further, flow cytometry experiments are performed to examine the effect of USP5 on lung cancer. Finally, the investigations in vivo are executed using the mice subcutaneous tumor model to identify the effect of USP5 in promoting lung cancer development.

**Results:**

Notably, USP5 is highly expressed in lung cancer, USP5 overexpression promoted the proliferation and migration in the lung cancer cell lines, H1299 and A549, while knockdown of USP5 inhibited these *via* regulating the PARP1-mediated mTOR signaling pathway. Furthermore, the subcutaneous tumors model was established in C57BL/6 mice, and the volume of subcutaneous tumors was significantly reduced after silencing USP5, while increased after USP5 overexpression and decreased significantly with shRARP1 treatment at the same time.

**Conclusions:**

Together, USP5 could promote the progression of lung cancer cells by mTOR signaling pathway and interacting with PARP1, indicating that USP5 may become a new target for lung cancer treatment.

## Background

In recent times, lung cancer has emerged as one of the fastest-growing malignant tumors in terms of an obvious increase in morbidity and mortality, seriously affecting people’s health and life safety. Notably, this dreadful cancer commonly occurs in elderly people over 50 years, with a male to female ratio of about 5:1 [1, 2]. Moreover, the clinical symptoms of lung cancer are relatively complex and closely related to tumor sites and pathological types. Lung cancer is often immature without any evident clinically relevant features and symptoms, which could be usually discovered with the physical examination. Moreover, the clinical pathogenesis of lung cancer is still unclear [3]. However, several studies indicated that lung cancer is related to various aspects, such as smoking, carcinogenic factors, chronic lung infection, genetic factors, and air pollution [4]. Currently, chemotherapy is prescribed to more than 90% of lung cancer patients as a systemic treatment, including advanced lung cancer patients. Although chemotherapy does not completely eradicate lung cancer, it only prolongs the survival rate and improves the quality of life [5, 6]. In this regard, it is obligatory to explicitly study lung cancer-related genes and molecular mechanisms, aiming to reveal the process and mechanisms of lung cancer origination and explore the effective treatment.

Over the past few decades, research studies demonstrated that mTOR is an important cellular signaling pathway involved in immunosuppression and protein synthesis [7]. mTOR is a family of filament/threonine kinases composed of mTORC1 and mTORC2. Indeed, the mTOR signaling pathway is generally activated in tumors, regulating cell growth, apoptosis, and autophagy. In this framework, mTOR enhances the efficiency of mRNA translation to promote protein synthesis towards the growth and division of cells [8, 9]. Ubiquitination is the post-translational modification of target proteins by ubiquitin molecules, restoring the stability of target proteins through cascading enzyme reactions [10, 11]. On the contrary, deubiquitination enzymes can specifically hydrolyze the peptide linkages, detain ubiquitin molecules from target proteins, and regulate cell protein degradation, as well as apoptosis. In this regard, ubiquitin-specific protease 5 (USP5), one of the important members of the deubiquitinase family, is involved in the activation of multiple signaling pathways, which regulate the biological activity of tumor suppressor genes, DNA repair enzymes, modified proteins, and other proteins, and thus participate in cell growth and division [12–15].

Recently, several studies reported that PARP (Poly ADP-ribose Polymerase), a DNA repair enzyme, plays an important role in DNA damage repair in most eukaryotic cells and the regulation mechanism of telomere structure of cancer cells [16, 17]. In addition, PARP1 is a cleavage substrate from caspase, a core member of cell apoptosis. In terms of DNA repair, PARP1 mainly affects repair function by DNA base excision, inhibits the transcription of damaged DNA, depletes energy in cells, and involves the transcriptional regulation of some genes. Notably, PARP1 is commonly used to repair single base breaks in DNA, restoring protein properties through covalent action [18]. Meanwhile, PARP1 can also cause cell death in cases of excessive DNA damage in a cell. Owing to these aspects, PARP1 inhibitors have been used in combination with cancer-killing drugs that partially cause DNA damage as a target for drug development research, effectively improving the efficiency of drugs to kill cancer cells [19]. Moreover, research has found that loss of PARP1 protein may increase the risk of cancers, such as lung cancer.

## Results

### USP5 is upregulated in lung cancer tissues

**Fig. 1:**
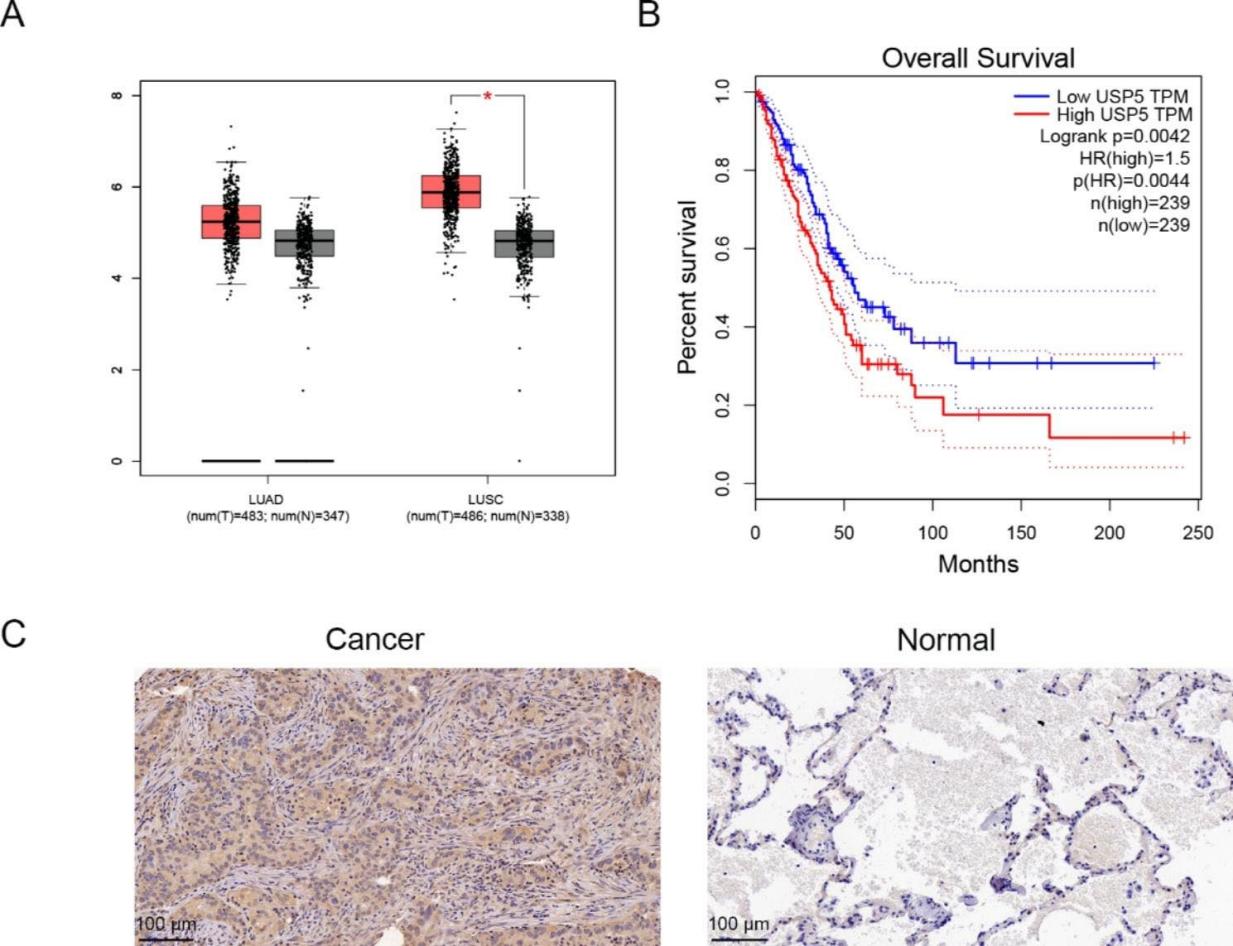
USP5 is related with the overall survival of lung cancer patients and high expressed in lung cancer tissues. (**A**) Expression of USP5 levels in lung cancer through GEPIA analysis based on the online TCGA database. (**B**) The overall survival rates of cells in correlation with the USP5 expression levels. (**C**) Immunohistochemical staining images showing the levels of USP5 protein in the corresponding lung cancerous and paracancerous tissues. * *P* < 0.05

To determine the role of USP5 in lung cancer development, we first evaluated the USP5 expression in lung cancer based TCGA database. The results indicated that the USP5 expression levels were significantly high, analyzed through GEPIA using the online TCGA database (Fig. 1A). The overall survival rate in the low USP5 level group was greater than that of the USP5 high expression group (Fig. 1B). In addition, immunohistochemical staining was employed to measure the expression of USP5 in lung cancerous and paracancerous tissues. It was observed that the levels of USP5 protein in lung cancer tissue were up-regulated than in corresponding paracancerous tissues (Fig. 1C; Table 1). These data demonstrated that the upregulation of USP5 is a frequent event in lung cancer and may play a key role in the prognosis of patients with lung cancer.

**Table 1:** The expression of USP5 in lung cancer and normal tissues

Type	USP5 expression		χ^2^	P-value
	High	Low		
Normal	6	19	11.54	0.0007
Lung cancer	18	7		

### USP5 promoted the progression of lung cancer cells in vitro and in vivo

**Fig. 2:**
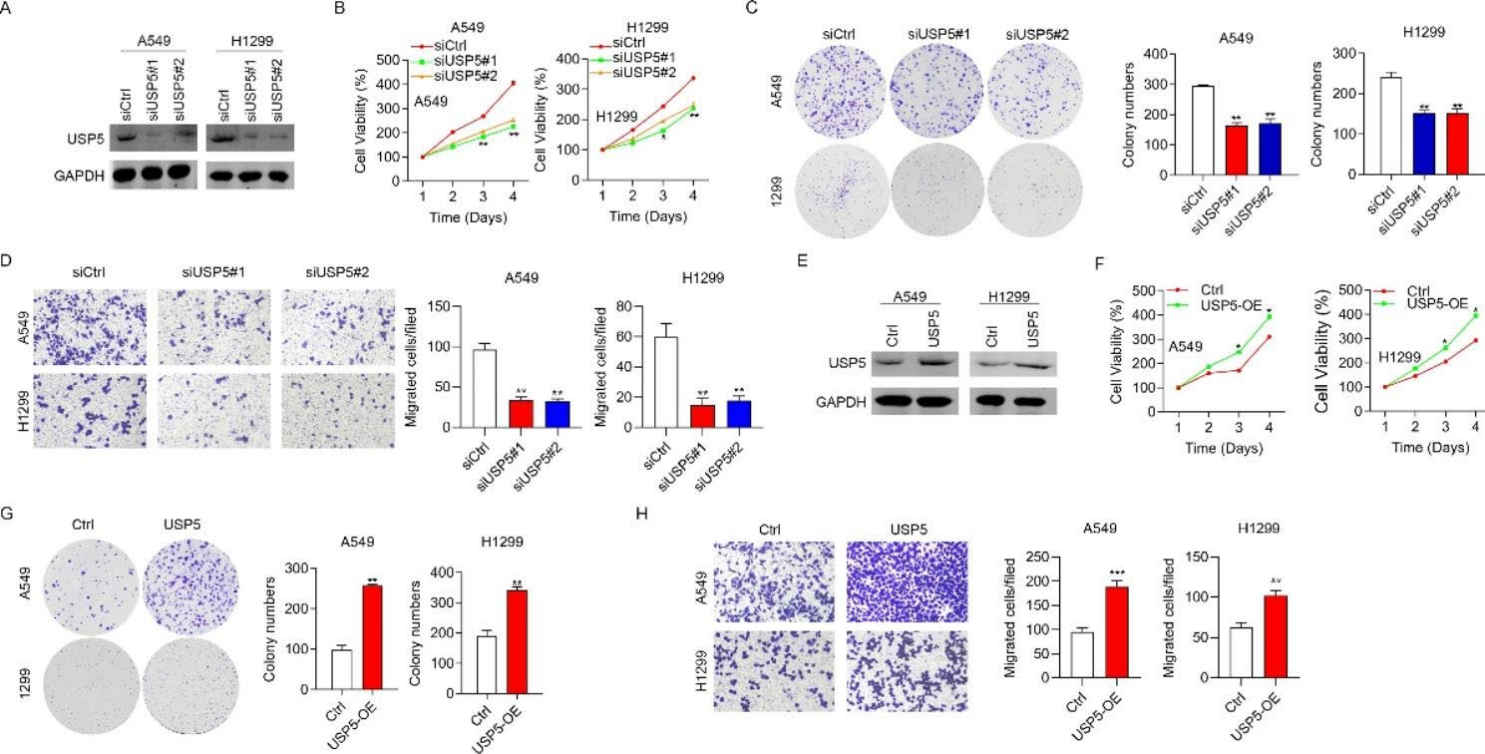
USP5 promotes H1299 and A549 cells proliferation and migration. (**A**) The expression levels of USP5 via RNA interference were checked by Western blotting. (**B**) MTT assay was performed to examine the cell viability after knocking down USP5. (**C-D**) Colony-forming assay represents the proliferation ability of H1299 and A549 cells after downregulation of USP5. (**E**) Ctrl and USP5 overexpressed H1299 and A549 cells were subjected to Western blotting analysis of USP5. (**F-H**) Proliferation, colony formation and migration abilities were analyzed in H1299 and A549 cells after over-expression of USP5. * *P* < 0.05, and ** *P* < 0.01

To evaluate the USP5 role in the lung cancer progression, the “gain of function” and “loss of function” strategies were used. The western blotting assay indicated that the USP5 was successfully knockdown in A549 and H1299c cells (Fig. 2A). MTT assay showed the cell expression of siUSP5#1 and siUSP5#2 groups was significantly suppressed compared with the siCtrl group (Fig. 2B) Further, the proliferation ability of the cells was measured by colony-forming assay, in which the proliferation ability was significantly reduced after down-regulation of the USP5 gene in H1299 and A549 cells (Fig. 2C). Similarly, the migration ability was also apparently reduced after down-regulation of the USP5 gene in H1299 and A549 cells (Fig. 2D). To further verify the role of USP5, a regression test was employed to up-regulate the USP5 expression, which was confirmed through western-blot (Fig. 2E). It was evident that the cell viabilities were promoted along with the up-regulation of the USP5 expression (Fig. 2F). Furthermore, the proliferation and migration abilities were significantly enhanced after over-expression of USP5 (Fig. 2G-H).

**Fig. 3:**
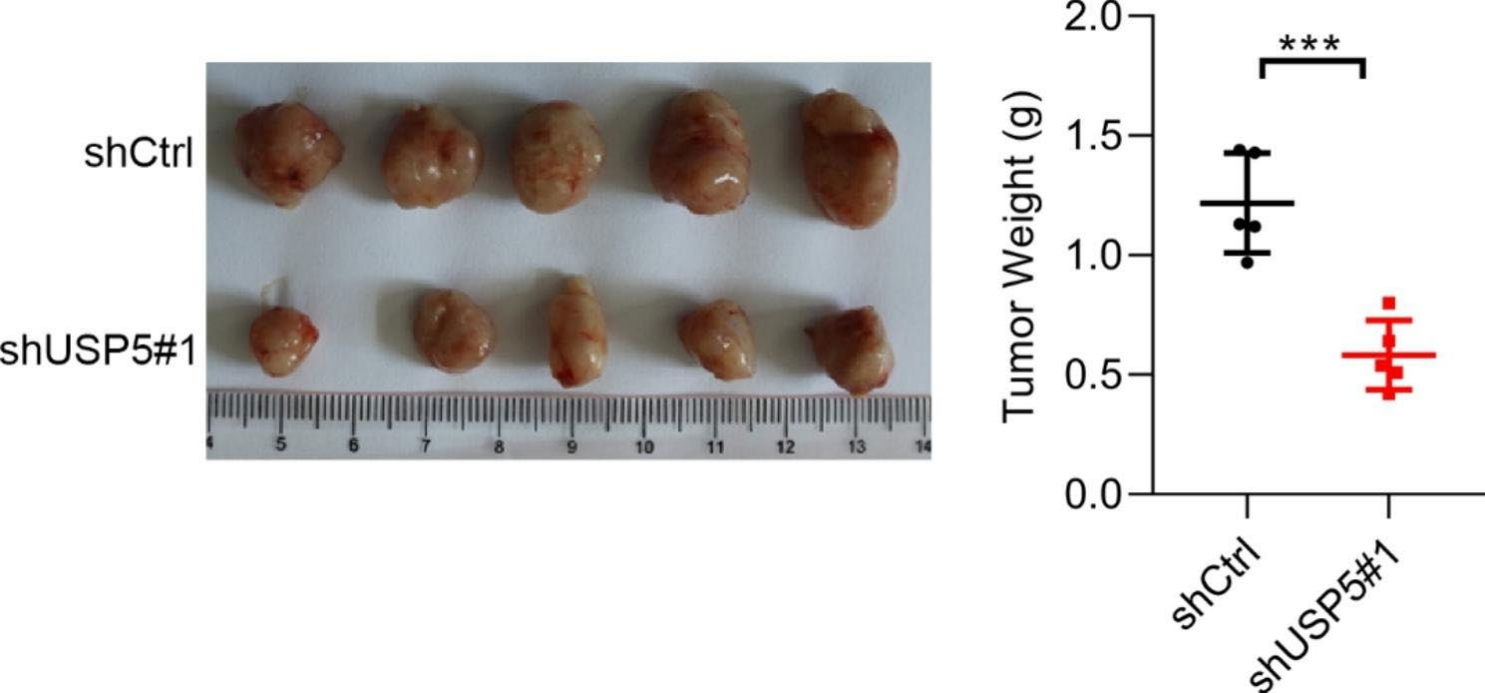
**USP5 knockdown suppressed the xenograft growth.** Downregulation of USP5 suppressed the xenograft growth and resulted in the apparent decrease in the xenograft weights. *** *P* < 0.001

Next, we established a subcutaneous transplant model in nude mice with USP5-knockdown A549 cells. We found that the silencing of USP5 suppressed the xenograft growth and resulted in the apparent decrease in the xenograft weights (Fig. 3). Taken together, these results indicated that USP5 possesses oncogenic activities in lung cancer.

### USP5 was regulated by mTOR signaling pathway

**Fig. 4:**
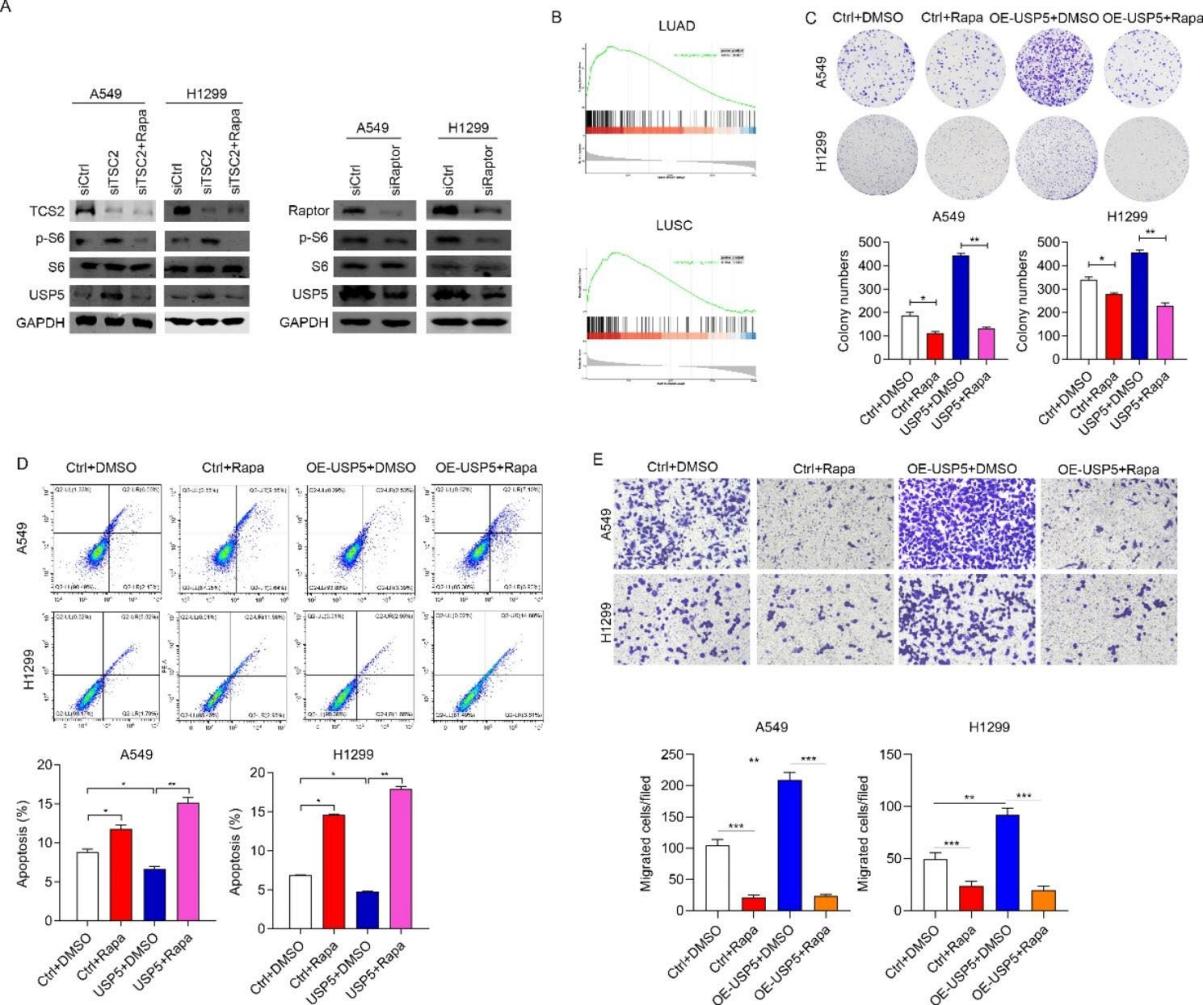
**mTOR activation of USP5 promotes the proliferation and migration of lung cancer cells. (**A) Left, siCtrl, siTSC2 and siTSC2 cells treated with rapamycin were subjected to Western blotting analysis of various proteins of mTOR markers and USP5. Right, siCtrl and siRaptor cells were subjected to Western blotting analysis of various proteins of mTOR markers and USP5. (**B**) GSEA analysis showed that USP5 high expression was correlated with high Mtorc1 activity in lung cancer patients. (**C-E**) The proliferation, apoptosis, and migration efficiencies of cells were measured by colony-forming assay, flow cytometry detection, and transwell chamber, respectively, in Ctrl and USP5 overexpressed cells treated with rapamycin. * *P* < 0.05, ** *P* < 0.01

To confirm whether USP5 was regulated by mTOR signaling, we activated mTOR signaling by the knockdown TSC2, and subsequently blocked the activation by Rapa. Similar with p-S6, a downstream protein of mTOR signaling, USP5 was significantly upregulated in siTSC2 group, while inhibited by Rapa (Fig. 4A). Moreover, we also detected the USP5 expression after mTOR signaling pathway inhibition through silencing Raptor. As expected, the USP5 protein level was decreased in siRaptor group (Fig. 4A). Next, we evaluated whether the expression of USP5 regulated by ontogenetic signaling. Based on TCGA database, we found that the expression of USP5 was positively correlated with mTOR signaling in both LUCS and LUAD samples (Fig. 4B). The above results indicated USP5 may be regulated by mTOR signaling. To confirm this conclusion. These results indicated that USP5 expression was regulated by mTOR signaling. Next, we explored the role of mTOR signaling in malignancy caused by USP5. To address this question, we detected the proliferation, apoptosis, and migration in USP5 overexpressed cells treated with Rapa or without Rapa. As showed in Fig. 4C, blockage of mTOR signaling pathway significantly reversed the USP5 promoted colony formation effect in A549 and H1299 cells. Additionally, the apoptosis rate was also marked increased by Rapa in USP5 overexpressed A549 and H1299 cells (Fig. 4D). Furthermore, blockage of mTOR signaling could significantly reduce migrated ability in USP5 overexpressed A549 and H1299 cells (Fig. 4E). Taken together, these results indicated that mTOR signaling is the upstream of USP5 and regulated the expression of USP5 in lung cancer.

### Interaction of USP5 with de-ubiquitinated PARP1

**Fig. 5:**
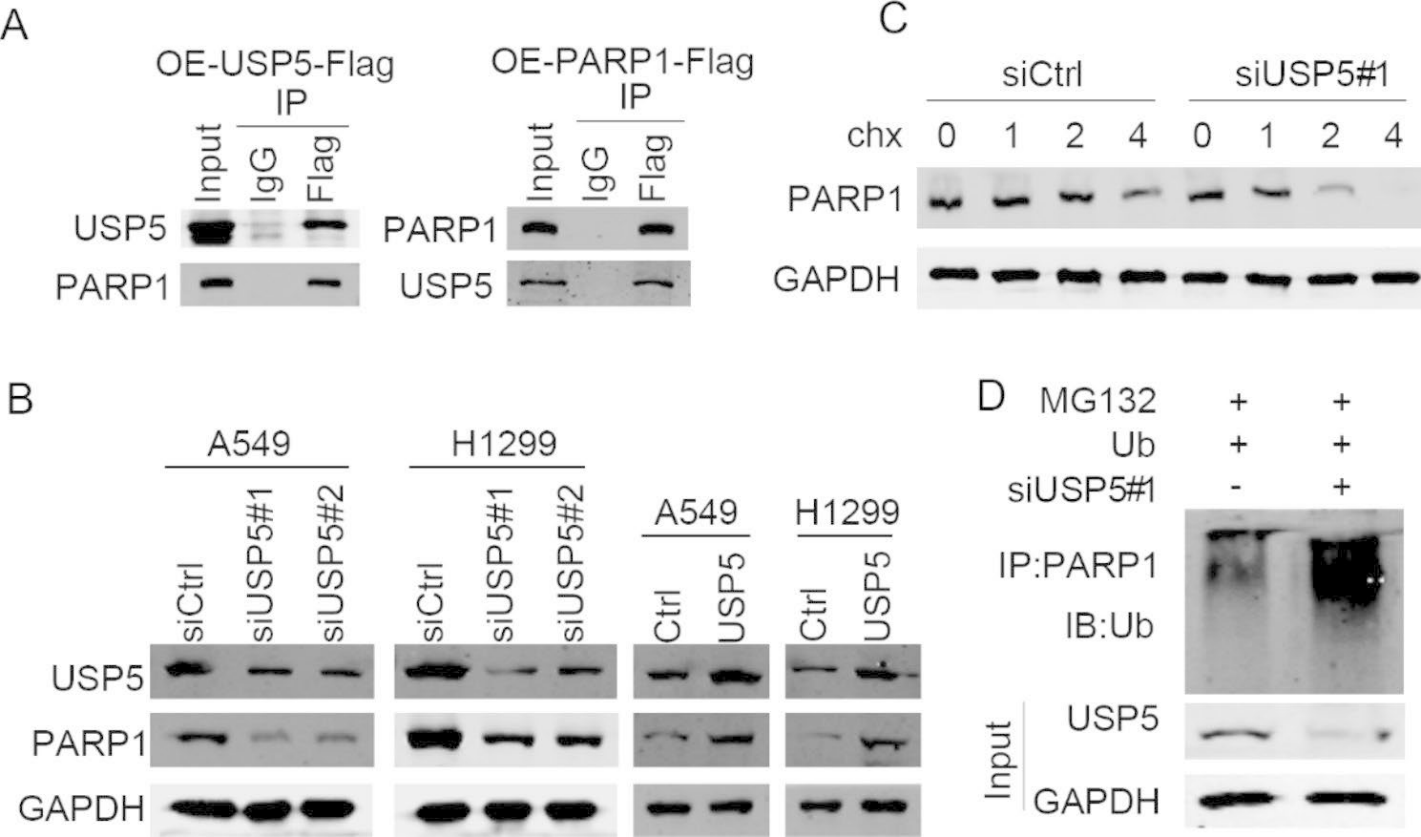
**USP5 promotes the progression of lung cancer by regulating PARP1. (A**) USP5-Flag and PARP1-Flag were overexpressed in HEK293 cells. The cell lysates were subjected to immunoprecipitation with IgG and Flag and immunoblotting analysis of USP5 and PARP1. (**B**) USP5 and PARP1 protein levels were detected by immunoblotting in USP5 overexpressed and knockdown cells. (**C**) siCtrl and siUSP5 cells were treated with cycloheximide and subjected to immunoblotting analysis of USP5. (**D**) Ubiquitin was overexpressing in siCtrl and siUSP5 cells which were treated with MG132. The cells were IP with PARP1 antibody. Ubiquitionation abundance was detected by immunoblotting

Initially, the co-immunoprecipitation approach was employed to confirm the relationship between USP5 and PARP1. Reciprocal IP results showed that USP5 interacted with PARP1 in A549 cells. In addition, USP5 overexpression upregulated PARP1, while its knockdown downregulated PARP1in lung cancer cells (Fig. 5A, B). To detect whether USP5 regulates the ubiquitination of PARP1, siCtrl and siUSP5 cells were treated with cycloheximide, a protein synthesis inhibitor. Immunoblotting results demonstrated that protein degradation was much more obvious in siUSP5 cells as compared with siCtrl cells (Fig. 5C). Lastly, we demonstrated that USP5 knockdown enhanced the ubiquitination levels of PAPR1 (Fig. 5D). Together, USP5 regulated the ubiquitination and expression of PARP1 through direct interaction.

### Effect of USP5 on the progression of lung cancer dependent on PARP1

**Fig. 6:**
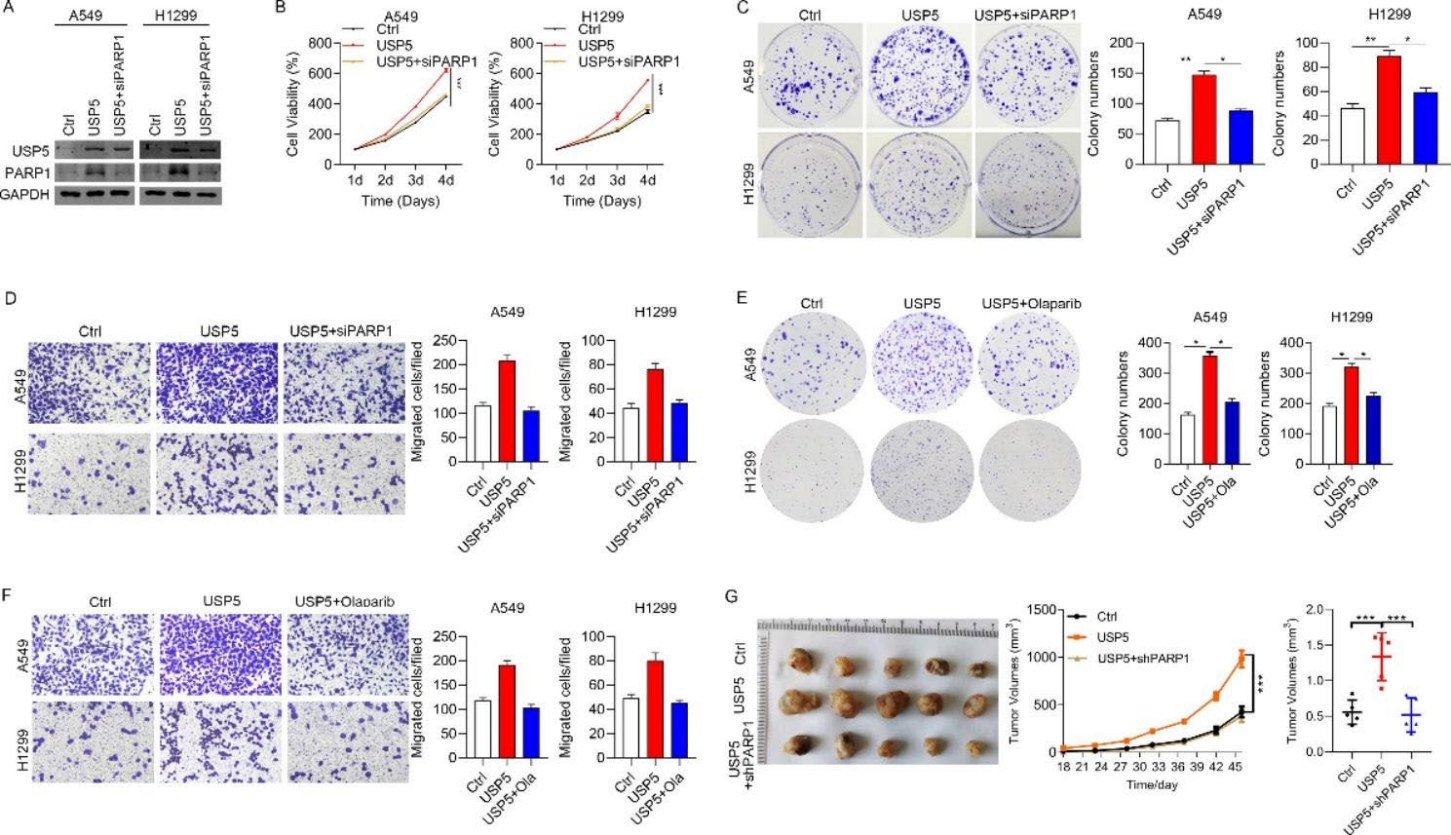
**USP5 promotes the progression of lung cancer by targeting PARP1 in vitro and vivo. (A**) Western blots showed that PARP1 was efficiently knocked down in USP5 overexpressed lung cancer cells. (**B-D**) Effect of PARP1 knockdown on the proliferation, colony formation, and migration abilities of USP5 overexpressing lung cancer cells. (**E-F**) Role of PARP1 inhibitors on colony formation, and migration abilities of USP5 overexpressing cells. (**G**) Effect of PARP1 knockdown on the volume of subcutaneous tumor in mice. * *P* < 0.05, ** *P* < 0.01, *** *P* < 0.001

To determine the effect of USP5 expression in lung cancer progression dependent on PARP1, H1299 and A549 cell lines were employed and treated with control, USP5, and USP5 with siPARP1, respectively. As shown in Fig. 6A, the lung cancer cells treated with USP5 could not effectively disregard the effects of PARP1 knockdown on their progression, inferring that the USP5 might function effectively combined with PARP1. However, the knockdown of PARP1 could significantly reduce the proliferation, colony formation, and migration abilities of these selected lung cancer cells (Fig. 6B-D). Further, the PARP1 inhibitors were co-cultured with cells to explore the effect of PARP1, in which the colony formation, and migration abilities were also reduced (Fig. 6E, F). Interestingly, the in vivo investigations showed that obliterating the effect of USP5 could significantly reduce the volume of subcutaneous tumors in mice (Fig. 6G).

## Discussion

Lung cancer is one of the most prevalent malignant tumors due to increased morbidity and mortality rates. As most of the tumor cells originate from the bronchial mucosal epithelium or gland, it is often known as primary bronchial lung cancer. In China, 0.8 million lung cancer patients are diagnosed with a 78% mortality rate each year [20, 21]. In recent times, it is alarming that the incidence and mortality rates of lung cancer have shown a dramatically rising trend in the past decade. Notably, the incidence rate of this lung cancer in males is more compared to those of females. Hitherto, more than half of lung cancer patients are over 70 years old and approximately 10% less than 40 years old. Although the pathogenesis of lung cancer is quite complex, it has been increasingly recognized that smoking is one of the biggest risk factors for lung cancer. Based on the pathology, lung cancer can be arbitrarily divided into two classes: the peripheral and central types, commonly referred to as adenocarcinoma and squamous cell carcinoma, respectively [22, 23]. One of the earlier symptoms is atypical, which may be manifested as an irritating dry cough, and there was no cognition to the complication. Further symptoms include hemoptysis, chest pain, fever, and weight loss, which could occur as the disease progresses. Accordingly, it is required to demonstrate the origination, classification, and development trend of the lung cancer disease in patients, towards reasonable and orderly treatment to improve the quality of life and prolong the life expectancy of the patients [24, 25]. Among various treatment options available, surgical treatment is the best choice for early lung cancer. However, more than 80% of the patients have been diagnosed with lung cancer in the advanced stage, coincidentally losing the opportunity for a precise and radical treatment. With an in-depth understanding of the mechanism of lung cancer, molecular targeted therapy has gained importance in treating the middle and advanced stages of lung cancer. In this study, we observed that the expression of USP5 promoted the progression of lung cancer cells by the mTOR signaling pathway and interacted with PARP1.

During the translation modification of proteins in the cancer cells, the deubiquitination enzymes specifically remove the ubiquitin-inhibiting substrates [26]. USP5 is a member of such ubiquitin-specific protease family, which can effectively remove the ubiquitin signals at the damaged sites of DNA double-strand and then effectively repair DNA [13]. In this framework, several research studies have shown that USP5 knockout can significantly increase the expression of P53 and its transcriptional activity[13, 27, 28]. In the study, it was observed that the level of USP5 protein in lung cancer tissues was higher than in the corresponding paracancerous tissues. Moreover, the proliferation ability was significantly reduced after down-regulation of the USP5 gene in the H1299 and A549 cells, significantly promoting after over-expression of USP5 levels. Accordingly, it could be concluded that the USP5 levels could substantially regulate the proliferation of tumor cells.

In addition, as p53 is closely related to the mTOR signaling pathway, we then examined the relationship between USP5 and the mTOR signaling pathway. mTOR is an atypical serine/threonine-protein kinase, one of the important members of the phosphatidylinositol-related kinase family. mTOR plays a tremendously important role in the proliferation, differentiation, and metastasis of cells as it is involved in gene transcription, protein translation and ribosome synthesis, and other biological processes [29–31]. In recent years, with the in-depth studies on the biological functions of mTOR, increased attention has been paid to exploring the relationship between mTOR and cell apoptosis, protein synthesis, and metabolism. Owing to these aspects, the mTOR signaling pathway has become a substantial target for cancer therapy [32]. In this regard, the Western blotting method was employed to validate that the USP5 expression levels could be up-regulated after mTOR signaling pathway activation in H1299 and A549 cell lines. Rapamycin, an mTOR signaling pathway inhibitor, was used to identify that the expression of USP5 was regulated by the mTOR signaling pathway.

PARP1, a DNA repair enzyme, was reported to regulate the mTOR signaling pathway [33, 34]. In the study, it was observed that the USP5 could directly bind to PARP1 by immunocoprecipitation. After the knockdown of PARP1, the effect of USP5-dependant progression in lung cancer cells will be terminated. Moreover, the relationship between USP5 and PARP1 was tested in the mice model with a subcutaneous tumor. Interestingly, it was observed that abolishing the effect of PARP1 could significantly reduce the volume of subcutaneous tumors in mice.

## Conclusions

In summary, it was observed that the USP5 was highly expressed in lung cancer tissues. Interestingly, the increased expression levels of USP5 protein had significantly promoted the progression of lung cancer cells by the mTOR signaling pathway and interacted with PARP1. Together, we believe that eliminating the effect of USP5 could delay the progression of lung cancer, becoming a potential target for lung cancer treatment.

## Methods

### Patients

The carcinomas and corresponding paracancerous tissues were collected from patients with lung cancer undergoing pneumonectomy at Peking Union Medical College Hospital. All subjects were informed of the scientific research project and signed the informed consent form. The study was approved by the Ethics Committee of Peking Union Medical College Hospital, Peking Union Medical College and Chinese Academy of Medical Sciences.

### Reagents

A549, H1299 cell lines were purchased from China Center for Type Culture Collection (Wuhan, China). DMEM/F12, high glucose medium, and fetal bovine serum (FBS) were purchased from Gibco/BRL Life Technologies (Carlsbad, USA). A colony-formation assay kit was obtained from KeyGEN BioTECH. Ltd. (Nanjing, China). Trizol was purchased from Beijing Solarbio Science and Technology Ltd. (Beijing, China). Primary antibodies against Beta-actin, GAPDH, USP5, mTOR were purchased from Santa Cruz Biotechnology Inc. (Dallas, USA). Primary antibodies against PARP1, TSC2, p-S6, T-S6, p-4EBP1, T-4EBP1 were obtained from Abcam Biotechnology (Cambridge, UK).

### Cell culture and passage

The cryopreserved tubes containing cells in liquid nitrogen were removed and placed in the thermostatic water bath set to 37℃. After shaking the cryopreservation tube to promote rapid thawing, the cells were then cultured with DMEM/F12 medium containing 10% FBS. Further, the cells were transferred to cell culture dishes and placed in an incubator (37℃ with 5% CO_2_) and cultured for 24 h. Then, a fresh medium was added to stop trypsin digestion. The cell suspension was transferred to a sterile centrifuge tube, and the supernatant was discarded by centrifugation. Finally, the cells were cultured with DMEM/F12 medium containing 10% FBS, and the cell suspension was uniformly transferred to the cell culture dishes and placed in an incubator.

### Protein extraction and Western blotting

Firstly, the cell protein was extracted using the mixture of PMSF (100×) with the protease inhibitor of RIPA lysate at the ratio of RIPA to PMSF as 1:100. Further, the cell protein concentration was measured by BCA working reagent and then added with 5x loading buffer in a water bath at 100℃ for 10 min. According to the manufacturer’s instructions, an equal amount of protein was added for electrophoresis and subsequently transferred to the PVDF membrane. Further, the membrane was sealed with 5% skim milk containing TBST at room temperature for 2 h. The membrane was washed thrice with TBST and incubated with the primary antibody for 12 h. Then, the membrane was incubated with the secondary antibody for 2 h. Finally, the ECL reagent (Beyotime Biotechnology, Shanghai, China) was employed for chemiluminescence development to monitor the blots.

### MTT assay

The cells at the logarithmic phase were collected and seeded into 96-well plates and incubated (37℃ with 5% CO_2_) overnight for better cell adherence. After adding 80 µl of fresh medium, 20 µl of the MTT working solution was added and cultured for 4 h. Then, 150 µl of dimethyl sulfoxide (DMSO) was added to each well, and the formazan crystals were completely dissolved by shaking at a low speed for 10 min. Finally, the absorbance values of each well were measured at 490 nm with the plate analyzer.

### Colony formation assay

The cells in the logarithmic growth phase were digested with 0.25% trypsin and suspended in DMEM/F12 medium containing 10% FBS. Then, the cell suspension was diluted gradient, and the cells of each group were seeded into a six-well plate and incubated. Further, the cells were fixed with 4% paraformaldehyde for 15 min. Then the fixing solution was removed and added GIMSA for the dyeing solution. The clone formation rate was calculated by observing under a microscope.

### Migration assay

Cells were cultured for 12 h with a serum-free medium and digested with 0.25% trypsin. After washing twice with PBS, the cells were suspended with a serum-free medium containing BSA. Then, 150 µl of cell suspension was added into the Transwell chamber and cultured at 37 ° C with 5% CO_2_ for 48 h. Further, the cells were stained with 0.1% crystal violet and decolorized with 33% acetic acid. After eluting the crystal violet completely, the cells were observed under a microscope.

### Apoptosis analysis

Flow cytometry was used to detect cell apoptosis. Initially, the cells at a logarithmic growth stage were collected and seeded at the density of 1 × 10^6^/mL. After being cultured for 72 h, 2 mL of cells from each group were collected. Further, the supernatant was discarded by centrifugation, washed with PBS, and fixed with 70% ethanol at 4 °C. After 24 h, the cells were added with propidium iodide (PI) and RNA enzyme A and stained at 4 °C for 30 min without light. The cell suspensions were stained with PI. Finally, the data were analyzed using FlowJo V10 software.

### Immunohistochemical staining

Initially, 3% H_2_O_2_ was added to the cultured cells and incubated at room temperature for 10 min to eliminate peroxidase activity. The cells were then washed with distilled water and immersed in PBS for 5 min. Further, 5% goat serum was added and incubated at room temperature for 10 min. Then, the cells were incubated in primary antibody for 2 h and followed by the secondary antibody for 30 min. Finally, the horseradish enzyme was added for 30 min followed by the chromogenic reagent.

### In vivo investigations

4–6 weeks old female Balb/c nude mice were obtained from Charles River Laboratories, and then adaptive feeding was employed for one week before the experiment. For USP5 knockdown assay, the mice were randomly divided into two groups, with 5 mice in each group; while for the rescue assay, the mice were randomly divided into three groups, with 5 mice in each group. A total of 100 µl cell suspension (5 × 10^7^ cells/ml) was injected for each mouse to establish a subcutaneous tumor mouse model. Further, the mice were sacrificed, and the subcutaneous tumors were collected and weighed at predetermined time intervals. The research was approved by the Ethics Committee of Peking Union Medical College Hospital, Peking Union Medical College and Chinese Academy of Medical Sciences.

### Statistical analysis

All the data were presented as the mean ± standard deviation (SD). The data comparisons between the groups were analyzed using t-test, and one-way analysis of variance (ANOVA) followed by Tukey’s honestly significant difference post-hoc test using SPSS19.0 and Prism9 software, considering P < 0.05 as a defined statistical significance.

## Data Availability

The datasets used and analyzed in this study are available from the corresponding author on reasonable request.

## Abbreviations

USP5: Ubiquitin-specific protease 5
PARP1: Poly ADP-ribose Polymerase
mTOR: Mammalian target of rapamycin
TSC2: Tuberous sclerosis complex 2
4EBP1: Eukaryotic translation initiation factor 4E binding protein 1
PMSF: Phenylmethanesulfonyl fluoride
TBST: Tris buffered saline tween
BSA: Bovine serum albumin
TCGA: The Cancer Genome Atlas
qRT-PCR: Quantitative real-time polymerase chain reaction
FBS: Fetal bovine serum
DMSO: Dimethyl sulfoxide
PI: Propidium iodide
